# Optimal cutoff value of the dry eye-related quality-of-life score for diagnosing dry eye disease

**DOI:** 10.1038/s41598-024-55358-1

**Published:** 2024-02-26

**Authors:** Xinrong Zou, Ken Nagino, Yuichi Okumura, Akie Midorikawa-Inomata, Atsuko Eguchi, Alan Yee, Keiichi Fujimoto, Maria Miura, Jaemyoung Sung, Tianxiang Huang, Kenta Fujio, Yasutsugu Akasaki, Shintaro Nakao, Hiroyuki Kobayashi, Takenori Inomata

**Affiliations:** 1 Department of Ophthalmology, Juntendo University Graduate School of Medicine, 2-1-1, Hongo, Bunkyo-ku, Tokyo, 113-0033 Japan; 2 Department of Ophthalmology, Fengcheng Hospital, Fengxian District, Shanghai, China; 3 Department of Hospital Administration, Juntendo University Graduate School of Medicine, Tokyo, Japan; 4 Department of Digital Medicine, Juntendo University Graduate School of Medicine, Tokyo, Japan; 5 AI Incubation Farm, Juntendo University Graduate School of Medicine, Tokyo, Japan

**Keywords:** Eye diseases, Corneal diseases, Epidemiology

## Abstract

This retrospective study aimed to determine the optimal cutoff values of the Dry Eye-Related Quality-of-Life Score (DEQS) questionnaire for diagnosing dry eye disease (DED) and classifying DED severities. Participants completed the DEQS questionnaire, the Japanese version of the Ocular Surface Disease Index (J-OSDI) questionnaire, and DED examinations. DED was diagnosed according to the 2016 Asia Dry Eye Society diagnostic criteria based on DED symptoms (J-OSDI ≥ 13 points) and tear film breakup time ≤ 5 s. Receiver operating characteristic (ROC) analysis was used to calculate the optimal cutoff values of the DEQS summary score for detecting DED and grading its severity. Among 427 patients, 296 (69.3%) and 131 (30.7%) were diagnosed with DED and non-DED, respectively. ROC analysis determined an optimal cutoff value of 15.0 points for DED diagnosis, with 83.5% sensitivity, 87.0% specificity, and an area under the curve of 0.915. The positive and negative predictive values for DEQS ≥ 15.0 points were 93.6% and 69.9%, respectively. DEQS cutoff values of 15.0, 20.0, and 26.8 points could be accepted for severity classification of DED subjective symptoms in clinical use and represent mild, moderate, and severe DED, respectively. Conclusively, the optimal cutoff values of DEQS enable DED detection and subjective symptom severity classification.

## Introduction

Dry eye disease (DED) is a common ocular surface disorder worldwide^1,2^. This multifactorial disease can lead to ocular surface damage and severely affect patients’ vision, quality of life, and work productivity^3,4^. These conditions are mainly caused by the instability of tear film homeostasis, which is the core pathogenesis of DED occurrence^5^. The assessment of the subjective symptoms of DED and dry eye examinations, primarily the tear film breakup time (TFBUT), constitute the key diagnostic elements of diagnosis criteria, such as the Tear Film and Ocular Surface Society and Asia Dry Eye Society^5,6^.

Subjective questionnaires continue to be important tools for assessing subjective symptoms of DED^7^. The Ocular Surface Disease Index (OSDI) questionnaire may be the most widely used in clinical research and screening; however, it does not cover all dry eye symptoms, such as foreign body sensation and health-related quality of life issues^7–9^. In contrast, the Dry Eye-Related Quality-of-Life Score (DEQS) questionnaire, developed in Japan in 2013, considers health-related quality-of-life issues^10,11^. The validity and reliability of the DEQS have been confirmed to evaluate the multifaceted effects of DED on patients’ daily lives, including ocular symptoms and mental health^9,11,12^. The DEQS questionnaire showed strong correlations with four subscales (ocular pain, near vision, distance vision, and mental health) of the National Eye Institute Visual Function Questionnaire 25^6^. Additionally, the DEQS was significantly correlated with the Japanese version of the OSDI (J-OSDI) questionnaire, with insignificant score differences^13^, suggesting that the DEQS questionnaire could be considered equivalent to the J-OSDI. However, different cutoff values of the DEQS summary score have been reported in previous studies^9,11^, as there is no optimal cutoff value for the DEQS questionnaire^14^. Additionally, the absence of a clear grading system could affect the classification of subjective symptoms, thereby affecting the treatment of diseases^15^. Thus, it was necessary to investigate the optimal cutoff values of the DEQS summary score based on subjective symptoms and TFBUT for DED detection and severity classification.

Accordingly, this study aimed to determine the optimal DEQS cutoff values for DED detection and symptom severity categorization by evaluating the sensitivity and specificity of the DEQS based on clinical symptoms and TFBUT. We believe that the DEQS questionnaire can be used more extensively for diagnosing DED in clinical practice, health check-up screening, and online medical services.

## Results

### Participants’ characteristics

A total of 427 individuals were enrolled; among them, 296 (69.3%) were diagnosed with DED, and 131 (30.7%) were diagnosed with non-DED according to the 2016 Asia Dry Eye Society diagnostic criteria. No significant differences in age and sex were observed between the non-DED and DED groups. The DED group had a significantly higher DEQS summary score and J-OSDI total score and lower TFBUT and maximum blink interval (MBI)^16,17^ than those in the non-DED group (Supplemental Table S1).

### Correlations between DEQS and other clinical assessment findings

We examined the relationship between the DEQS summary score and other clinical assessment findings (J-OSDI, TFBUT, corneal and conjunctival fluorescein staining (CFS), Schirmer I test (SΙT), and MBI) using Pearson’s correlation test. Pearson correlation analysis showed that the DEQS summary score was significantly positively correlated with the J-OSDI total score (*P* = 0.874) and negatively correlated with the MBI (*P* = − 0.236) (Table 1).

**Table 1:** Correlations between the DEQS and other clinical parameters in included participants.

Clinical items	DEQS	J-OSDI	TFBUT	CFS	SIT	MBI
DEQS	1.000					
J-OSDI	0.874***	1.000				
TFBUT	− 0.030	− 0.011	1.000			
CFS	0.039	0.047	− 0.334***	1.000		
SIT	− 0.063	− 0.105	0.143	− 0.176***	1.000	
MBI	− 0.236***	− 0.213***	0.338***	− 0.186***	0.129	1.000

Pearson correlation coefficients were estimated among the DEQS summary score, J-OSDI total score, TFBUT, CFS, SIT, and MBI.

*P* values were considered statistically significant at * < .05, ** < .01, and *** < .001.

*DEQS* Dry Eye–Related Quality-of-Life Score; *J-OSDI* Japanese version of the Ocular Surface Disease Index; *TFBUT* tear film breakup time; *CFS* corneal and conjunctival fluorescein staining; *SIT* Schirmer I test; *MBI* maximum blink interval.

### ROC curves of the DEQS for DED detection

**Figure 1:**
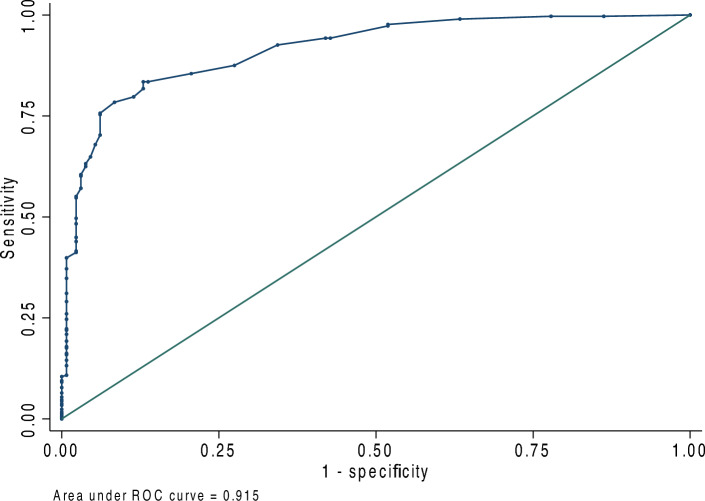
Receiver operating characteristic (ROC) analysis of the Dry Eye-Related Quality-of-Life Score for dry eye disease diagnosis. The area under the curve calculated based on the ROC was 0.915. The optimal cutoff value for the Dry Eye-Related Quality-of-Life Score was 15.0 points, which yielded a sensitivity of 83.5% and a specificity of 87.0%.

In the receiver operating characteristic (ROC) curve for the sensitivity and specificity of the DEQS, the area under the curve (AUC) calculated based on ROC was 0.915. The optimal cutoff value of the DEQS for DED detection was 15.0 points, which yielded a sensitivity of 83.5% and a specificity of 87.0% (Fig. 1).

### DEQS cutoff values for DED severity classification via ROC analysis

The DEQS cutoff values for DED severity classification were calculated via ROC analysis corresponding to the J-OSDI score categorization. The optimal cutoff values are listed in Table 2.

**Table 2:** DEQS cutoff values for DED severity classification via ROC analysis.

DED classification	J-OSDI	DEQS	AUC	Sensitivity (%)	Specificity (%)
Mild DED	13.0	15.0	0.937	83.6	91.9
Moderate DED	23.0	20.0	0.946	80.8	94.5
Severe DED	33.0	26.8	0.940	88.7	86.0

*DEQS* Dry Eye–Related Quality-of-Life Score; *DED* dry eye disease; *ROC* receiver operating characteristic; *J-OSDI* Japanese version of the Ocular Surface Disease Index; *AUC* area under the curve.

### Precision rate detected by the DEQS at the cutoff value of 15.0 points

Table 3 shows the precision rates of diagnosis using TFBUT with the DEQS. The positive and negative predictive values for DEQS ≥ 15 points were 93.6% (247/264) and 69.9% (114/163), respectively (Table 3). The sensitivity and specificity were 83.5% (247/296 individuals) and 87.0% (114/131 individuals), respectively (Table 3).

**Table 3:** Precision rate detected DED by the DEQS at the cutoff value of 15 points.

Groups	Non-DED	DED	Total
DEQS < 15 points (%)	114 (87.0)	49 (16.5)	163 (38.2)
DEQS ≥ 15 points (%)	17 (13.0)	247 (83.5)	264 (61.8)
Total	131	296	427

*DEQS* Dry Eye–Related Quality-of-Life Score; *DED* dry eye disease.

### Characteristics of participants detected by DEQS at different cutoff values

The DEQS measurements of participants with different DED severities were compared with those without DED. Based on the DEQS cutoff value of 15 points, the mild DED group (DEQS ≥ 15.0 to < 20.0 points) had significantly higher J-OSDI (*P* < 0.001) and lower MBI (*P* < 0.001) than the non-DED group (DEQS < 15 points) (Table 4). Similarly, the moderate DED group (DEQS ≥ 20.0 to < 26.8 points) also showed significantly higher J-OSDI (*P* < 0.001) and lower MBI (*P* < 0.001) than the non-DED group (Table 4). A similar result was shown in the severe DED group (DEQS ≥ 26.8 points), which had a higher J-OSDI (*P* < 0.001) and a lower MBI (*P* < 0.001) than the non-DED group (Table 4).

**Table 4:** Comparison of the characteristics between the DED and non-DED groups detected by the DEQS at different cutoff values.

	Non-DED (DEQS < 15.0)	Mild DED (DEQS ≥ 15.0 to < 20.0)	*P* value	Moderate DED (DEQS ≥ 20.0 to < 26.8)	*P* value	Severe DED (DEQS ≥ 26.8)	*P* value
Age (years), mean ± SD	61.8 ± 17.0	60.1 ± 15.4	.273	59.8 ± 15.1	.152	59.3 ± 15.5	.094
Sex, number (%)			.380		.100		.094
Male	29 (17.8)	38 (14.4)		32 (13.2)		24 (12.6)	
Female	134 (82.2)	226 (85.6)		211 (86.8)		166 (87.4)	
BCVA (logMAR), mean ± SD	− 0.019 ± 0.106	− 0.032 ± 0.191	.324	− 0.025 ± 0.197	.321	0.002 ± 0.214	.189
IOP (mmHg), mean ± SD	13.7 ± 3.0	14.0 ± 2.7	.241	14.1 ± 2.7	.167	14.0 ± 2.6	.354
DEQS (0–100 points), mean ± SD	6.7 ± 4.2	42.3 ± 19.6	< .001	44.5 ± 18.9	< .001	50.5 ± 17.0	< .001
J-OSDI (0–100 points) mean ± SD	10.3 ± 9.0	44.1 ± 19.8	< .001	46.1 ± 19.2	< .001	51.0 ± 18.1	< .001
TFBUT (seconds), mean ± SD	1.7 ± 1.1	1.7 ± 1.2	.856	1.7 ± 1.3	.466	1.7 ± 1.2	.973

Data are presented as mean ± standard deviation or n (%). *P* values were estimated using a t-test for continuous variables and χ^2^ test for categorical variables.

*SD* standard deviation; *DED* dry eye disease; *DEQS* Dry Eye-Related Quality-of-Life Score; *BCVA* best-corrected visual acuity; *IOP* intraocular pressure; *J-OSDI* Japanese version of the Ocular Surface Disease Index; *TFBUT* tear film breakup time; *CFS* corneal and conjunctival fluorescein staining; *SIT* Schirmer I test; *MBI* maximum blink interval.

## Discussion

DED is a highly prevalent chronic condition of the ocular surface. The DEQS questionnaire correlates well with the severity of subjective symptoms and effects of DED on daily life^18^. However, the DEQS questionnaire has no optimal cutoff values for DED diagnosis and severity classification, which limits its use in the clinical setting. Thus, we aimed to detect DED and categorize the severity of the subjective symptoms of DED with optimal DEQS cutoff values using a hospital-based, cross-sectional, observational method. This study yielded a DEQS cutoff value of 15.0 points for DED diagnosis. Further, DEQS cutoff values of 15.0, 20.0, and 26.8 points could be adopted for the severity grading of subjective symptoms in clinical use. Notably, the DEQS with optimal cutoff values could be used in clinical studies for DED diagnosis and severity classification.

Different cutoff values of the DEQS have been reported in previous studies^9,11^. In a study conducted in Thailand, DED was suspected if the DEQS cutoff value was > 18.33 (AUC, 0.897; sensitivity, 90.0%; specificity, 76.7%)^9^. Their research adopted the following diagnostic criteria: ocular symptoms (OSDI ≥ 13 points) and tear film abnormality (TFBUT ≤ 5 s or SIT with anesthesia < 5 mm)^9^. Another study using a combination of the DEQS questionnaire and strip meniscometry score reported an optimal cutoff value of 15.0 points for the DEQS questionnaire and a strip meniscometry score < 5 mm (AUC, 0.904; sensitivity, 79.4%; specificity, 90.6%)^11^. In the current study, we explored the optimal DEQS cutoff value for DED diagnosis, which was DEQS ≥ 15.0 points combined with TFBUT ≤ 5 s (AUC, 0.915; sensitivity, 83.5%; specificity, 87.0%). Additionally, we discovered that the positive and negative predictive values of DEQS ≥ 15.0 points for predicting DED were 93.6% and 69.9%, respectively. In this study, we observed a limited number of false positives and false negatives due to score differences between DEQS and J-OSDI. DEQS includes questions related to depressive symptoms and eye problems associated with prolonged mobile phone screen use, whereas J-OSDI addresses environmental factors and the impact of DED on night driving. These differences in the questionnaire items that contribute to the scoring systems may have influenced the score variations between DEQS and J-OSDI^13^. However, despite these differences, the accuracy of DED diagnosis using a DEQS cutoff of ≥ 15 points remained consistently high. Hence, a DEQS questionnaire cutoff value of 15.0 points was deemed suitable for DED diagnosis.

To our knowledge, this is the first study to report the optimal cutoff values of the DEQS for the severity classification of subjective symptoms of DED. DEQS cutoff values of 15.0, 20.0, and 26.8 points in our study corresponded to the respective J-OSDI total scores of 13.0, 23.0, and 33.0 points, which are the suggested cutoff scores for mild, moderate, and severe degrees of DED on the J-OSDI scale^19^. Among the four groups classified by the above-mentioned optimal DEQS cutoff values, MBI values displayed a gradual downward trend (13.3 ± 7.4, 10.6 ± 6.8, 10.3 ± 6.6, and 10.0 ± 6.5 in DEQS < 15, ≥ 15.0 to < 20.0, ≥ 20.0 to < 26.8, and ≥ 26.8 groups, respectively). Hence, the descending tendency observed in the MBI values could show the classification effect of the DEQS optimal cutoff values proposed in this study. Notably, the DEQS cutoff value of 25.0 points is very close to the DEQS cutoff value of 26.8 points, as determined by the Youden index (0.741 vs. 0.747) (Supplemental Table S2). Therefore, considering the ease of use in a clinical setting, DEQS values of 15.0, 20.0, and 25.0 points could be proposed as the optimal cutoff values for DED severity stratification. Categorizing the severity of DED subjective symptoms is beneficial for patients’ self-management of DED and for clinicians to diagnose and treat DED more efficiently. As a result, medical resources can be used more effectively.

Here, when the DEQS cutoff value was set as 15.0 points, the DEQS ≥ 15.0 to < 20.0 group had a significantly lower MBI (10.6 ± 6.8 vs. 13.3 ± 7.4 seconds, *P* < .001) than the DEQS < 15.0 group. Similar results were also presented in the DEQS ≥ 20.0 to < 26.8 (10.3 ± 6.6 vs. 13.3 ± 7.4 seconds, *P* < .001) and DEQS ≥ 26.8 (10.0 ± 6.5 vs. 13.3 ± 7.4 seconds, *P* < .001) groups, with lower MBI than that of the DEQS < 15.0 group. MBI is a simple method for DED screening and was positively correlated with TFBUT in a previous study^16^. In our study, MBI was significantly associated with TFBUT. Recent epidemiological surveys have shown that shorter TFBUT is the most common manifestation of DED in clinical practice^20–22^. However, no significant difference was discovered in the TFBUT value between the DEQS < 15.0 group and the other three groups due to low TFBUT values. DED could be diagnosed using the DEQS with an optimal cutoff value of 15.0 points based on the MBI differences between the two groups. Thus, a DEQS cutoff value of 15.0 points is optimal for identifying DED using the MBI screening method.

The DEQS questionnaire possesses good internal consistency, test–retest reliability, discriminant validity, and responsiveness to change^9,10^. The optimal cutoff values for the DEQS obtained through our hospital-based, cross-sectional study, combined with a TFBUT value ≤ 5 s, enabled DED diagnosis and symptom severity classification with high sensitivity, specificity, and AUC. Additionally, the DEQS optimal cutoff values corresponded well with the proposed J-OSDI cutoff values for classifying DED severity. Therefore, considering the comprehensive nature of the DEQS compared with that of traditional dry eye-related questionnaires, the DEQS with the proposed optimal cutoff values for DED detection and severity classification may be better suited for wider implementation. Early diagnosis, together with timely treatment, could relieve dry eye symptoms and improve the quality of life of patients with DED, reducing the socioeconomic burden on society as a whole.

This study had some limitations. First, the data were collected using a Japanese questionnaire and exclusively from a single hospital in Tokyo, Japan. This could introduce selection bias, and as a result, the findings from this study may not be readily applicable to broader Asian populations. Second, the participants were older (60.3 ± 16.0 years), and there were more female participants, possibly due to the higher DED prevalence in older and female populations. Finally, the parameters of dry eye examinations, including TFBUT, SIT, and CFS, did not exhibit significant differences between these groups (Supplemental Table S3). Since our study only included individuals whose dry eye subjective symptoms had improved with eye drops and classified them into the non-DED group, these population characteristics had a potentially negative impact on the recorded dry eye test values in our study^23^. However, the MBI values showed a gradual downward trend in these groups, which could still reflect the DEQS capability to classify DED severity.

In conclusion, the present study illustrated the optimal cutoff values of the DEQS for DED detection and DED severity categorization in clinical activities. The combination of TFBUT and the DEQS with an optimal cutoff value for DED diagnosis generated high sensitivity and specificity. The optimal cutoff values of the DEQS for DED severity classification based on the J-OSDI standard also had high sensitivity and specificity via ROC analysis. Thus, the current study demonstrates the potency and feasibility of the DEQS for DED identification and severity classification in clinical diagnosis and health examination screening.

## Methods

### Study design and population

This study was a retrospective, hospital-based, cross-sectional, observational study. Outpatients who visited the Department of Ophthalmology at Juntendo University Hospital in Tokyo, Japan, were included in the study between September 2017 and September 2021. The enrolled patients comprised individuals with DED and other concomitant ocular diseases, all of whom underwent comprehensive ophthalmological assessments, including dry eye examinations. DED was diagnosed according to the Asia Dry Eye Society 2016 diagnostic criteria based on the presence of DED symptoms (J-OSDI ≥ 13 points as positive) and a TFBUT ≤ 5 s^5^. Patients with a history of eyelid disorder, ptosis, mental disease, Parkinson’s disease, severe ocular allergy diseases, those immediately after surgery, or any other disease that could affect blinking, as per previous research^16,17^, were excluded. The participants completed various assessments and ophthalmic examinations, including the DEQS questionnaire, J-OSDI questionnaire, slit-lamp microscopy, TFBUT, CFS, SIT, and MBI^16^.

### Ethical approval

This study was approved by the Independent Ethics Committee of Juntendo University Faculty of Medicine (approval number: E22-0365-H01) and was performed in accordance with the Declaration of Helsinki. The requirement for written informed consent was waived owing to the retrospective nature of the study by the Independent Ethics Committee of Juntendo University Faculty of Medicine; thus, the study was carried out using the opt-out method on our hospital website.

### DEQS questionnaire

The DEQS questionnaire primarily assesses the impact of DED on the patients’ quality of life^10^. It consists of 15 items that evaluate DED symptoms and how they have affected the subjects’ daily lives over the past week. All items in the DEQS questionnaire were divided into two sections: six questions regarding bothersome ocular symptoms and nine questions regarding the impact of DED on daily life. Columns A and B present the frequency and severity of each question, respectively. In column A, respondents provided a rating of the frequency of each symptom using a 5-point scale, ranging from “none of the time” (0 points) to “all the time” (4 points). A frequency score of 1–4 prompted the interviewee to move to column B, where they rated the degree of severity on a 4-point scale. Consequently, the DEQS score, ranging from 0 to 100, was calculated using the following formula: (sum of the degree scores for all questions answered) × 25/(total number of questions answered), with higher scores indicating increased severity of DED symptoms and a greater impact on daily life.

### J-OSDI questionnaire

The J-OSDI questionnaire is a translated Japanese version of the OSDI^24^. The reliability and validity of the DED diagnosis have been demonstrated in our previous studies^24,25^. The J-OSDI contains three subscales of 12 questions in total: ocular symptoms (three questions), vision-related functions (six questions), and environmental triggers (three questions). The participants were evaluated via graded symptoms on a 5-point scale from 0 points (none of the time) to 4 points (all the time). The J-OSDI total score, ranging from 0 to 100 points, was calculated by multiplying the sum score of all questions answered by 25 and dividing it by the total number of questions answered (N/A is selected when the question is not applicable). According to the J-OSDI total score, patients were stratified into four subgroups: normal (score, 0–12), mild (score, 13–22), moderate (score, 23–32), and severe (score, 33–100) symptoms^19,24^. The J-OSDI total score was positively associated with DED severity and impact on activities of daily living.

### Ocular examination procedures and clinical assessments

TFBUT was measured according to a standard procedure^6^. Ocular surface damage should be avoided, and the effect on tear volume and TFBUT should be minimized; therefore, fluorescein should be instilled at the outer canthus after removing excess saline on the strip. The participants were instructed to blink thrice to ensure satisfactory mixing of the dye with tears. The period between the last blink and the onset of the first dark spot on the cornea was recorded using a stopwatch. The mean values of three measurements were used. A cutoff value of TFBUT ≤ 5 s was used to diagnose DED^5^; the eye with the lower TFBUT value was used in the current study.

CFS was categorized based on the van Bijsterveld grading system^26^, and the ocular surface was divided into three regions: the nasal bulbar conjunctiva, the temporal bulbar conjunctiva, and the cornea. Each area was assessed on a scale of 0–3, with 0 indicating no staining and 3 indicating confluent staining; the maximum possible score was 9. The CFS assessment was conducted using reference diagrams from the van Bijsterveld grading system, as previously described in studies^27–29^.

MBI was defined as the length of time that the participants could keep their eyes open before blinking during each test^16,17^. MBI was calculated twice using a stopwatch under slit-lamp microscopy with the light off to protect the patient from glare. If MBI exceeded 30 s, it was recorded as 30.

After completion of all other examinations, SIT was performed without topical anesthesia. The strips for SIT were placed in the outer third of the temporal lower conjunctival fornix for 5 min. The strips were then removed, and the length of the wet filter paper (mm) was noted.

### Statistical analysis

The unpaired t-test was used for continuous variables, and the χ^2^ test was used for categorical variables. Pearson correlation coefficients were estimated for the DEQS summary score, J-OSDI total score, TFBUT, CFS, MBI, and SIT.

The ROC curve was created by calculating the sensitivity and specificity to determine the optimal cutoff values of the DEQS summary score for detecting DED and establishing normal, mild, moderate, and severe DED severity categories. The optimal cutoff value for the DEQS was determined when the Youden index, which is the sum of sensitivity and specificity minus one, was maximized^30^. The accuracy of DED detection using the calculated cutoff value of the DEQS was evaluated in terms of sensitivity, specificity, positive predictive value, and negative predictive value. Participants’ characteristics across DED severity categories, classified using the DEQS cutoff values, were compared to evaluate their validity.

Data are shown as the mean ± standard deviation or proportions (percentages). *P* values < 0.05 were considered statistically significant. Statistical analysis was performed using Stata version 17.1 (StataCorp, College Station, TX, USA).

### Supplementary Information

Supplementary Tables.

## Data Availability

The authors confirm that the data supporting the findings of this study are available within the article and/or its supplementary materials.
