# Supplementary material for: Optimal cutoff value of the dry eye-related quality-of-life score for diagnosing dry eye disease
Source: Sci Rep. 2024 Feb 26;14:4623. doi: 10.1038/s41598-024-55358-1 (PMC10897401; doi:10.1038/s41598-024-55358-1)
Supplement: Supplementary file 1 — Supplementary Tables. [file 41598_2024_55358_MOESM1_ESM.pdf]

Optimal Cutoff Value of the Dry Eye-Related Quality-of-Life Score for Diagnosing Dry Eye Disease

Xinrong Zou, Ken Nagino, Yuichi Okumura, Akie Midorikawa-Inomata, Atsuko Eguchi, Alan Yee, Keiichi Fujimoto, Maria Miura, Jaemyoung Sung, Tianxiang Huang, Kenta Fujio, Yasutsugu Akasaki, Shintaro Nakao, Hiroyuki Kobayashi, Takenori Inomata

SUPPLEMENTAL TABLE S1. Study population demographics and clinical characteristics

Characteristics	Non-DED (n = 131)	DED (n = 296)	Total (n = 427)	*P* value
Age (years), mean±SD	59.5 ± 17.9	61.3 ± 15.1	60.7 ± 16.0	.276
Sex, number (%)	131 (30.7)	296 (69.3)	427 (100)	.320
Male	24 (18.3)	43 (14.5)	67 (15.7)	
Female	107 (81.7)	253 (85.5)	360 (84.3)	
BCVA (logMAR), mean±SD	–0.031 ± 0.094	0.000 ± 0.011	-0.009 ± 0.164	.072
IOP (mmHg), mean±SD	13.8 ± 2.9	14.0 ± 2.8	13.9 ± 2.8	.547
DEQS summary score (0-100 points), mean±SD	8.5 ± 9.3	37.7 ± 22.0	28.7 ± 23.3	<.001
J-OSDI total score (0-100 points), mean±SD	7.8 ± 10.5	41.5 ± 19.6	31.2 ± 23.3	<.001
TFBUT (seconds), mean±SD	2.1 ± 1.8	1.5 ± 0.8	1.7 ± 1.2	<.001
CFS score (0-9 points), mean±SD	2.9 ± 2.6	3.3 ± 2.7	3.1 ± 2.6	.168
SIT (mm), mean±SD	7.8 ± 7.8	7.1 ± 7.3	7.3 ± 7.4	.361
MBI (seconds), mean±SD	14.4 ± 8.0	10.4 ± 6.4	11.6 ± 7.2	<.001
MGD, yes (%)	23 (17.6)	73 (24.7)	96 (22.5)	.105

Data are presented as mean ± standard deviation or n (%). *P* values were estimated using an

unpaired t-test for continuous variables and χ^2 test for categorical variables.
SD, standard deviation; DED, dry eye disease; BCVA, best-corrected visual acuity; IOP, intraocular pressure; DEQS, Dry Eye-Related Quality-of-Life Score; J-OSDI, Japanese version of the Ocular Surface Disease Index; TFBUT, tear film breakup time; CFS, corneal fluorescein staining; SIT, Schirmer I test; MBI, maximum blink interval; MGD, meibomian gland dysfunction.

SUPPLEMENTAL TABLE S2. Youden index at different DEQS cutoff values

Cutoff value	DEQS cutoff value	AUC	Sensitivity (%)	Specificity (%)	Youden index (sensitivity + specificity − 1)
Dry eye diagnosis (J-OSDI ≥13 and TFBUT ≤5 seconds)	15.0	0.915	83.5	87.0	0.705
J-OSDI (0-100 points), severity classification					
≥13 (mild)	15.0	0.937	83.6	91.9	0.755
≥23 (moderate)	20.0	0.946	80.8	94.5	0.753
≥33 (severe)	26.8	0.940	88.7	86.0	0.747
≥33 (severe)[a]	25.0	0.940	92.1	82.0	0.741

[a]The DEQS cutoff value of 25.0 was recommended to replace 26.8 for ease of use because it was extremely close to the DEQS cutoff value of 26.8 on account of the Youden index.
DEQS, Dry Eye–Related Quality-of-Life Score; AUC, area under the curve; J-OSDI, Japanese version of the Ocular Surface Disease Index; TFBUT, tear film breakup time.

SUPPLEMENTAL TABLE S3. Systemic disease characteristics of the study population

Characteristics	Non-DED	DED	P value	Total
Sex, number (%)				
Male	24 (18.3)	43 (14.5)	0.320	67 (15.7)
Female	107 (81.7)	253 (85.5)		360 (84.3)
Age (years)	61.3 ± 15.1	61.3 ± 15.1	0.276	60.7 ± 16.0
MGD, yes (%)	23/131 (17.6)	73/296 (24.7)	0.105	96/427 (22.5)
Eye drops use, yes	87/115 (75.7)	246/287 (85.7)	0.016	333/402 (82.8)

(%)				
Hypertension, yes (%)	34/120 (28.3)	75/269 (27.9)	0.927	109/389 (28.0)
Diabetes, yes (%)	13/119 (10.9)	34/268 (12.7)	0.624	47/387 (12.1)
Collagen disease, yes (%)	56/123 (45.5)	127/275 (46.2)	0.904	183/398 (46.0)
Sjogren's syndrome, yes (%)	27/122 (22.1)	78/273 (28.6)	0.181	105/395 (26.6)
Rheumatoid arthritis, yes (%)	9/121 (7.4)	23/270 (8.5)	0.719	32/391 (8.2)
Other collagen disease, yes (%)	31/123 (25.2)	57/270 (21.1)	0.367	88/393 (22.4)
Asthma, yes (%)	11/77 (14.3)	23/163 (14.1)	0.971	34/240 (14.2)
Mental disease, yes (%)	6/116 (45.2)	29/260 (11.2)	0.065	35/376 (9.3)

Data are presented as mean ± standard deviation or n (%). P values were estimated using a t-test for continuous variables and χ^2 test for categorical variables.

DED, dry eye disease; MGD, meibomian gland dysfunction.
